# Preliminarily exploring the intraoperative ultrasonography characteristics of patients with degenerative cervical myelopathy

**DOI:** 10.1186/s12891-024-07601-z

**Published:** 2024-07-12

**Authors:** Wenfen Liu, Jiachun Li, Tao Shu, Qiao Ji, Xianxiang Wang, Renjie Li, Yajuan Sui, Danni He, Zuofeng Xu

**Affiliations:** 1 Department of Ultrasound, The Seventh Affiliated Hospital of Sun Yat-Sen University, #628 Zhenyuan Road, Shenzhen, 518100 Guangdong China; 2 Department of Orthopaedics, The Seventh Affiliated Hospital of Sun Yat-Sen University, #628 Zhenyuan Road, Shenzhen, 518100 Guangdong China

**Keywords:** Cervical myelopathy, Intraoperative ultrasonography characteristics, Decompression, Laminoplasty, Spinal cord compression

## Abstract

**Background:**

How to quickly read and interpret intraoperative ultrasound (IOUS) images of patients with degenerative cervical myelopathy (DCM) to obtain meaningful information? Few studies have systematically explored this topic.

**Purpose:**

To systematically and comprehensively explore the IOUS characteristics of patients with DCM.

**Materials and methods:**

This single-center study retrospectively included patients with DCM who underwent French-door laminoplasty (FDL) with IOUS guidance from October 2019 to March 2022. One-way ANOVA and Pearson’s /Spearman’s correlation analysis were used to analyze the correlations between the cross-sectional area of the spinal cord (SC) and individual characteristics; the relationships between the morphology, echogenicity, pulsation, decompression statuses, compression types of SC, location of the spinal cord central echo complex (SCCEC) and the disease severity (the preoperative Japanese Orthopedic Association score, preJOA score); the difference of the spinal cord pulsation amplitude(SCPA) and the SCCEC forward movement rate (FMR) between the compressed areas(CAs) and the non-compressed areas (NCAs).

**Results:**

A total of 38 patients were successfully enrolled (30 males and 8 females), and the mean age was 57.05 ± 10.29 (27–75) years. The cross-sectional area of the SC was negatively correlated with age (*r* = − 0.441, *p* = 0.006). The preJOA score was significantly lower in the heterogeneous group than in the homogeneous group (*P* < 0.05, *p* = 0.005). The hyperechoic area (HEA) was negatively while the SCCEC FMR was positively correlated with the preJOA score (*r* = − 0.334, *p* = 0.020; *r* = 0.286, *p* = 0.041). The SCCEC FMR and SCPA in CAs were significantly greater than those in NCAs (*p* < 0.05, *p* = 0.007; *P* < 0.001, *P* = 0.000).

**Conclusion:**

The cross-sectional area of the SC decreases with age in adults. More changes in intramedullary echogenicity and less moving forward of the SCCEC often indicate poor SC status, and the SCCEC FMR and SCPA are more pronounced in CAs.

**Supplementary Information:**

The online version contains supplementary material available at 10.1186/s12891-024-07601-z.

## Introduction

Degenerative cervical myelopathy (DCM) is an umbrella term for a series of diseases that lead to chronic spinal cord (SC) compression and injury and are characterized by cervical spondylotic myelopathy, ossification of the posterior longitudinal ligament, degenerative disc disease and so on. DCM is a progressive, degenerative spine condition and is the leading cause of SC dysfunction worldwide [1, 2]. Even if symptoms are mild at the time of diagnosis, early surgical treatment is still recommended [3, 4]. Timely surgical decompression can prevent further SC deterioration and facilitate the recovery of neurological function for the majority of DCM patients [2, 5].

Cervical laminoplasty is a common surgical procedure for the treatment of DCM because of its advantages in expanding the spinal canal and preserving the posterior structure of the cervical spine. For patients with multilevel DCM, the preferred cervical laminoplasty method is French-door laminoplasty (FDL), which involves opening a “door” on the cervical posterior midline to achieve symmetrical enlargement of the cervical canal [6, 7]. However, during laminectomy, surgeons can assess only the morphology and motion of the posterior SC. It is difficult to fully evaluate the relationship between the anterior SC and the anterior compression element, whether decompression is sufficient, and the change of the intramedullary echo for even experienced surgeons due to the limited visual field [8–10]. Associated miscalculations may eventually lead to postoperative radiculopathy, persistent symptoms, poor neurologic recovery, or functional deterioration [9]. Therefore, a way is needed to help surgeons eliminate visual blindness to avoid such misjudgments during surgery.

Normally, the spinal canal is surrounded by bony structures, so the internal structure of the spinal canal cannot be clearly visualized via ultrasound. However, in cervical laminoplasty, especially in the FDL, after the spinous process is symmetrically opened, the shielding bone is removed, and 0.9% normal saline (NS) is used to fill the cavity to expel the air, creating an excellent acoustic window for ultrasound [8, 11]. Then, the dura mater, pia mater, subarachnoid space, morphology of the SC, positional relationship between the SC and anterior compression element, movement of the SC, intramedullary echo and position of the spinal cord central echo complex (SCCEC) can be distinctly revealed via intraoperative ultrasonography (IOUS) [12–14].

IOUS has long been used in spinal surgery since it was first reported in the 1980s [15, 16]. Due to the inherent advantages of ultrasound, especially it can achieve real-time dynamic imaging [17, 18]. In recent decades, IOUS has become an indispensable tool for surgeons to visualize structures beyond their visual field in cervical laminoplasty [9, 19]. The application of IOUS has mainly focused on the correlation between the parameters of IOUS and preoperative or postoperative MRI [20–22] and on the predicting postoperative SC function based on SC morphology, echo, pulsation, SC state after decompression and the location of compressors [10, 14, 19, 23–25]. However, most of the literature has focused on a single perspective. There is no consensus regarding which indicators are meaningful in the assessment of SC function. For surgeons and sonographers, especially those who lack relevant experience in IOUS, quickly interpreting ultrasound images to obtain useful information is highly important. To the best of our knowledge, there have been no published studies summarizing the characteristics of IOUS in patients with DCM. The aim of this study was to systematically and comprehensively explore the IOUS characteristics of patients with DCM; these findings could be helpful for both sonographers and surgeons in terms of more quickly recognizing and understanding IOUS images. Furthermore, these findings could be helpful for surgeons in terms of expanding the surgical field of view, assessing the status of the SC and making better targeted clinical decisions.

## Materials and methods

This study followed the principles outlined in the Declaration of Helsinki and was approved by the Seventh Affiliated Hospital of Sun Yat-sen University Ethics Committee. Complete informed consent was obtained from all participants in the study.

### Participants

The inclusion criteria were as follows: (a) adult age, (b) had multilevel DCM (≥ 3) confirmed by MRI, (c) underwent FDL with IOUS guidance, and (d) underwent surgery performed by the same team of surgeons in our hospital from October 2019 to March 2022. The exclusion criteria for patients were as follows: (a) a history of another spinal disorder, neurological injury, infection, tumor, or rheumatoid arthritis and (b) incomplete imaging data or poor image quality.

### Clinical assessments

The neurological function of all patients was evaluated separately by two orthopedic surgeons with more than 10 years of clinical experience according to the Japanese Orthopedic Association (JOA) score. The JOA score consists of 6 domains: motor function in the upper extremities, motor function in the lower extremities, sensory function in the upper extremities, sensory function in the trunk, sensory function in the lower extremities, and bladder function. The minimum total score is 0, and the maximum score is 17. The total score was used as the preJOA score [26–28]. Clinical data were obtained from medical records (Table 1).

**Table 1:** Clinical assessment data

Basic data	Results
Number of cases	38
Sex (male/female)	30/8
Age (years)*	57.05 ± 10.29
Height (meters)*	1.63 ± 0.12
Weight (kilograms)*	67.75 ± 11.95
BMI*	25.06 ± 2.69
preJOA score*	10.75 ± 2.87

*The data are presented as the mean ± standard deviation

### Surgical technique and IOUS

FDL was performed on all patients by the same surgical team via Kurokawa’s method, with modifications [22, 29]. After the spinous process was split in the middle and the spinal canal was opened, the surgical field was exposed, and 0.9% NS was injected into the spinal canal to provide a good acoustic window for IOUS. A linear array probe (4–12 MHz, M9 Expert; Mindray Medical International Limited, Shenzhen, China) was selected. The probe was coated with a sterile coupler and wrapped in a sterile sleeve. The surgeon held the probe to effectively control the force, avoiding additional pressure on the SC. The morphology, echo, activity and adjacent structures of the SC in compressed areas (CAs) and adjacent noncompressed areas (NCAs) were continuously and dynamically observed on the sagittal and transverse planes. M-mode ultrasound was used to observe the spinal cord pulsation amplitude (SCPA) in sagittal sections. More than 10 s of dynamic images were required for each plane for subsequent analysis. Images were recorded and stored as a digital movie file (avi). The whole ultrasound examination process was controlled within 10 min. According to the ultrasound findings, surgeons could fully understand the state of the SC after decompression and estimate the degree of neurological impairment to further optimize the surgical procedure. If there was residual compression, it was necessary to further expand the spinal canal until complete decompression was achieved. Typically, IOUS should be used only once after decompression; if further decompression is needed, IOUS should be used again after decompression, and the latter result is the final result.

### IOUS assessment

The IOUS characteristics were analyzed via dynamic video of the SC recorded by IOUS after decompression. All sonograms and videos were evaluated independently by two sonographers with more than 10 years of clinical experience who were blinded to the results of the clinical evaluation. All the data were measured using ImageJ software (version 1.53f51) at least three times. When there was a dispute between the two sonographers, a third sonographer, also with more than 10 years of experience, participated in the evaluation. When 2 or more sonographers made the same decision, the agreed upon result was adopted.

### SC morphology

**Fig. 1:**
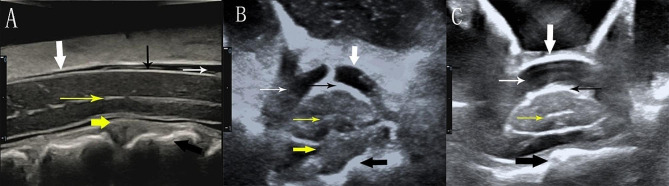
Intraoperative sagittal and cross-sectional sonograms of the SC. (**A**) Sagittal image. (**B**) Transverse image of the CA. (**C**) Transverse image of the NCA. The distance from 0 to 1 on the scale represents 1 centimeter. The spinal dura mater (bold white arrow), pia mater (thin black arrow), subarachnoid space (thin white arrow), SCCEC (thin yellow arrow), anterior compression element (bold yellow arrow), and posterior margin of the cervical vertebra (bold black arrow) could all be clearly visualized. Area_compressed_ was measured on transverse images in the CA (**B**); Area_free_ was measured on transverse images in the NCA (**C**)

The cross-sectional area of the SC was measured in the most severely compressed area (Area_compressed_) and in a noncompressed area (Area_free_) at least 1 vertebra away from the former. Referring to the previously described method of measuring the SC compression index on MRI and IOUS [30, 31], we selected the cross-sectional area instead of the anteroposterior diameter and calculated the spinal cord compression ratio (SCCR) according to the following formula: SCCR = Area_compressed_/Area_free_ (Fig. 1). The correlation between the SCCR and preJOA score was also analyzed. As Area_free_ nearly represents the actual thickness of the SC, the relationships between Area_free_ and patient sex, age, height, weight, and BMI were analyzed.

### Intramedullary echo changes

The patients were divided into homogeneous and heterogeneous groups according to whether the SC exhibited abnormal echogenicity (hyperechogenicity and anechogenicity) on IOUS.

**Fig. 2:**
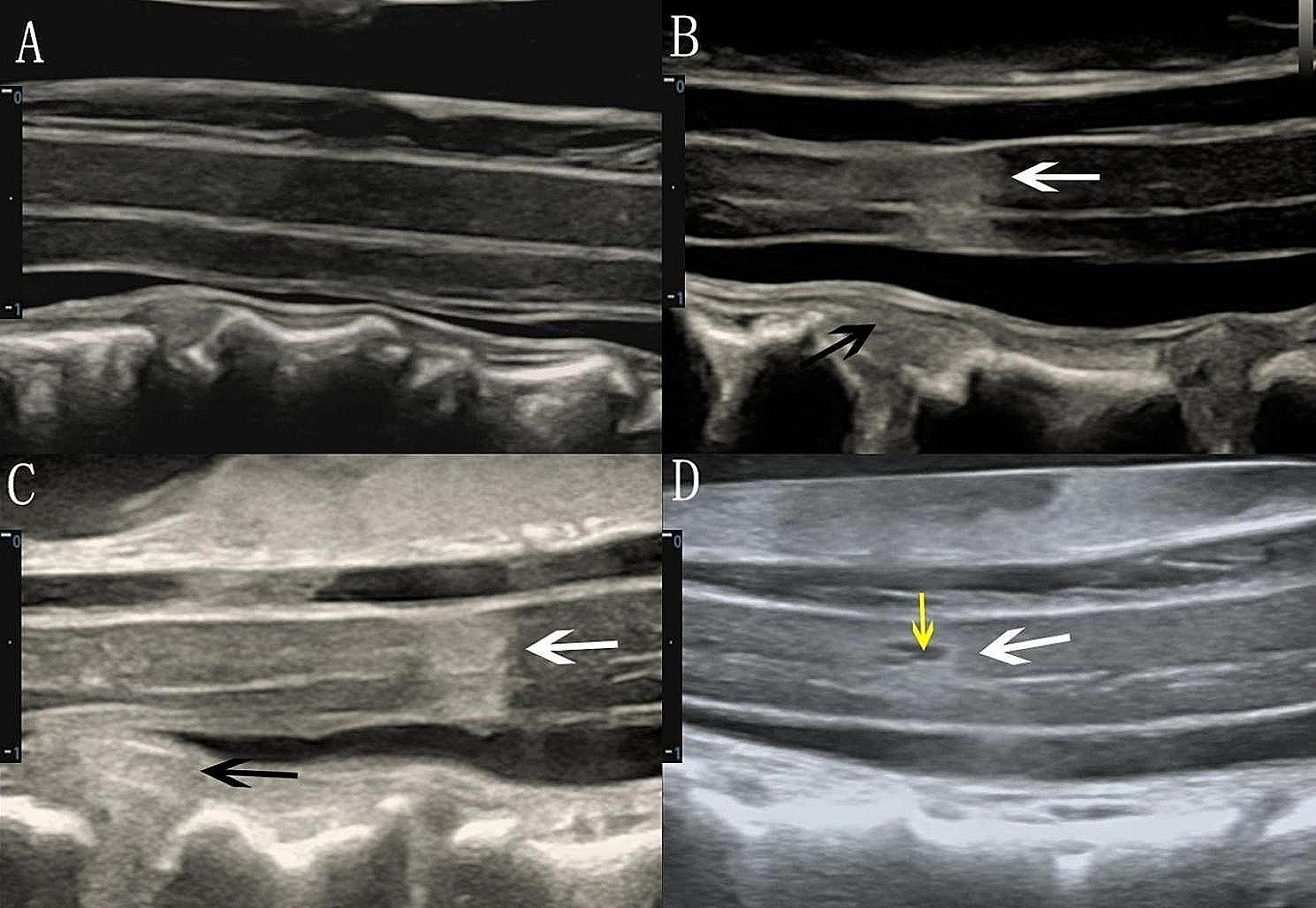
Intramedullary echo changes on the sagittal view. (**A**) Homogeneous intramedullary echo. (**B**) Heterogeneous intramedullary echo (white arrow) near the anterior compression element (black arrow). (**C**) Heterogeneous intramedullary echo (white arrow) away from the anterior compression element (black arrow). (**D**) Hyperechogenicity in the SC (white arrow) and anechogenicity (yellow arrow) in the area of hyperechogenicity The distance from 0 to 1 on the scale represents 1 centimeter

Abnormal echogenicity mainly manifested as hyperechogenicity in the parenchyma, while anechogenicity could be observed in the areas of hyperechogenicity in a few patients. Then, the area of hyperechogenicity (hyperechoic area, HEA) was measured according to a previously reported method with some modifications [32, 33] (Fig. 2). Instead of using software to calculate the pixel sum of the region of interest (ROI), we traced the area of hyperechogenicity directly on the two-dimensional image.

### SC pulsation

Patients were divided into the SC pulsation group (Video 1) and the SC nonpulsation group (Video 2) according to the presence or absence of SC pulsation on the sagittal plane.

**Fig. 3:**
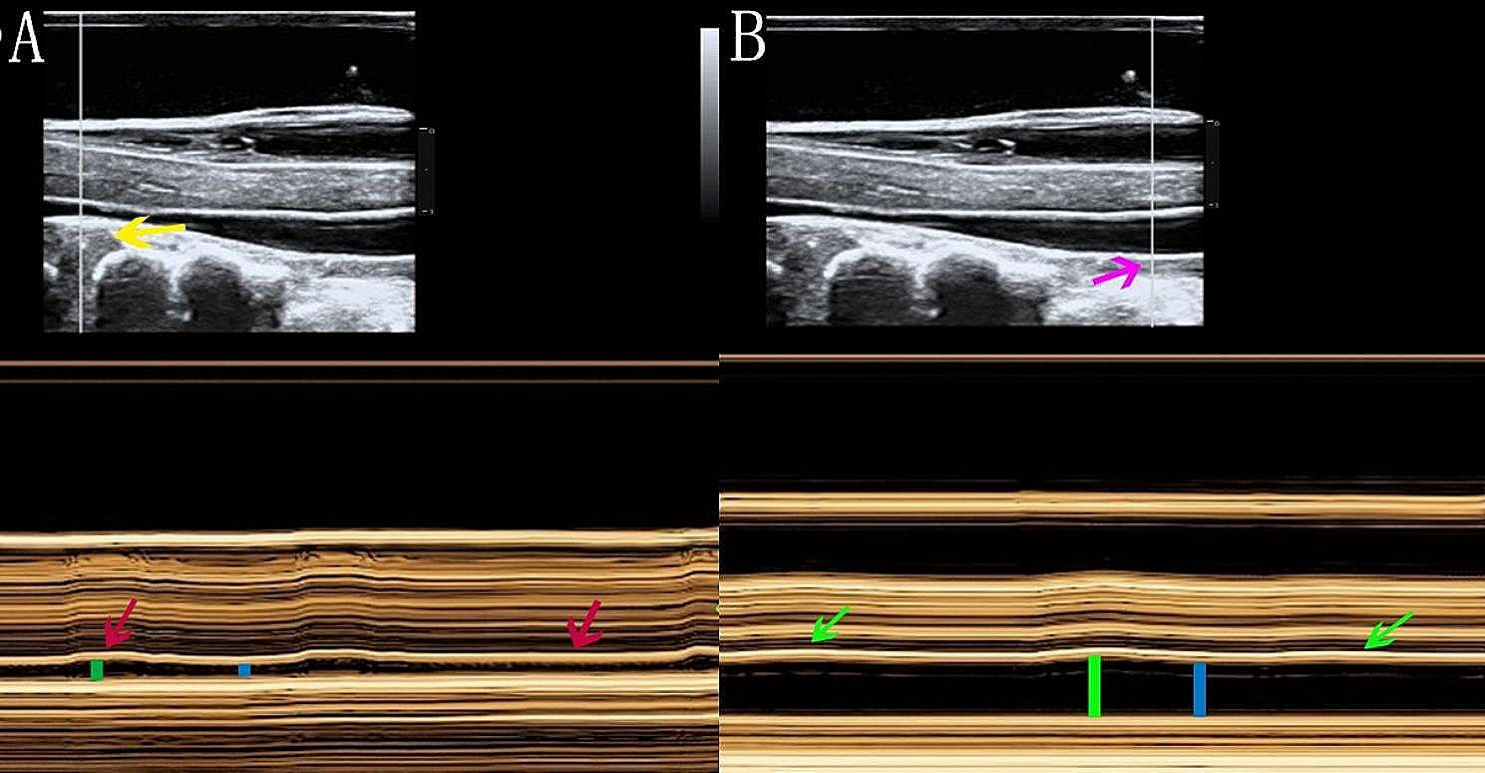
SCPA in the CA (**A**) and NCA (**B**) visualized via M-mode ultrasound. (**A**) Sample line passing through the CA (yellow arrow). (**B**) Sample line passing through the NCA (pink arrow). The wavy lines graphically depict the SCPA in the CA (red arrow) and NCA (green arrow). SCPA = length of the green line minus the length of the blue line. The distance from 0 to 1 on the scale represents 1 centimeter

In the pulsation group, the SCPA was defined as the maximum distance (from the target to the posterior longitudinal ligament) minus the minimum distance (Fig. 3). The SCPA was measured at the anterior edge of the SC at the most severely compressed segment and at an adjacent noncompressed segment at least 1 vertebra away.

### Status of decompression

IOUS video analysis allowed the decompression status to be divided into three types according to the relative positions of the anterior compression element and the anterior edge of the SC [24, 25, 31]. The differences in the preJOA score among the three decompression statuses were also determined.

Type 1. Continued separation: The SC completely floated in the cerebrospinal fluid (Video 3).

Type 2. Contact and separation: As the SC pulsated, the anterior edge contacted and separated from the anterior compression element (Video 4).

Type 3. Continued contact: The anterior compression element and the anterior edge of the SC were in constant contact (Video 5).

### Position of compression

**Fig. 4:**
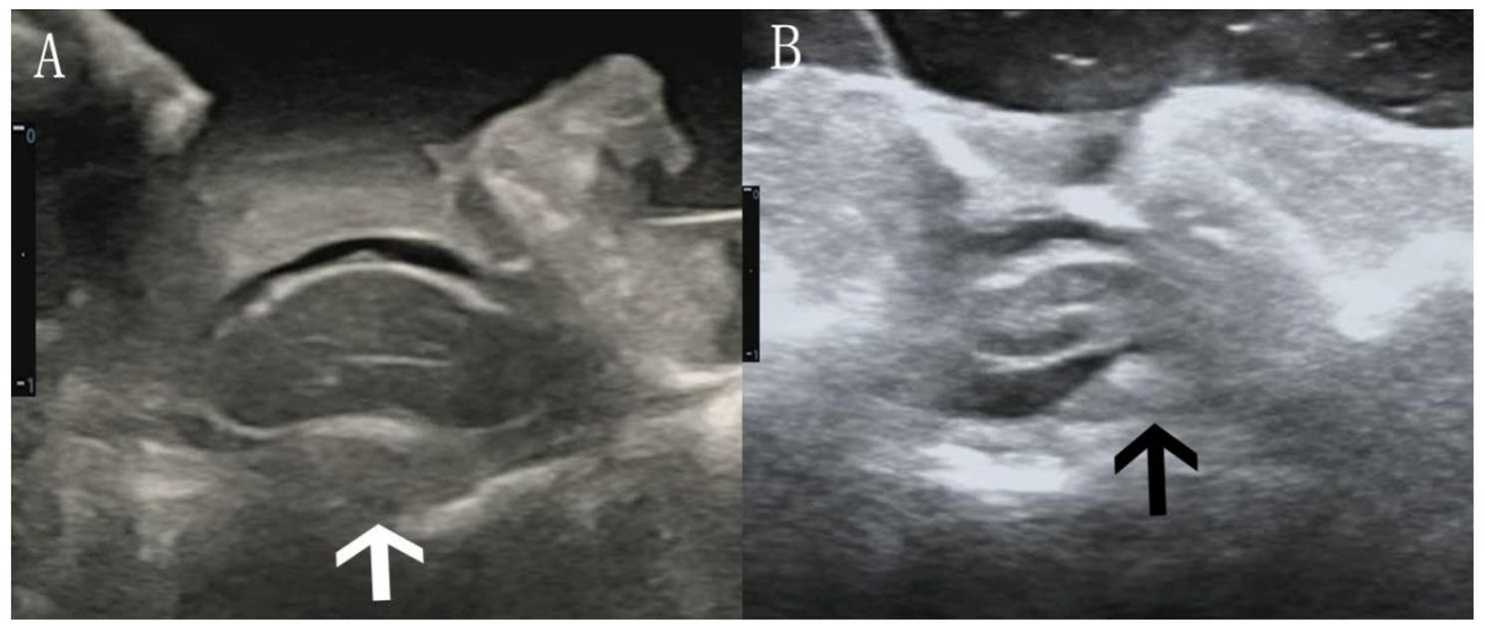
Cross-sectional view of different types of compression. (**A**) Anterior compression element located in the center of the SC (white arrow). (**B**) Anterior compression element located lateral to the SC (black arrow). The distance from 0 to 1 on the scale represents 1 centimeter

Patients were divided into central and lateral compression groups according to the position of the anterior compression element according to a previously reported method, with some modifications [23]. In this study, we merged the whole width of the SC compression and central SC compression into central type. Compression of the anterior spinal artery was more common in the central group, while it was not significantly or even not compressed in the lateral group (Fig. 4).

The symptom severity (preJOA score) was compared between patients with different compression agent types.

### Forward shift of the SCCEC

**Fig. 5:**
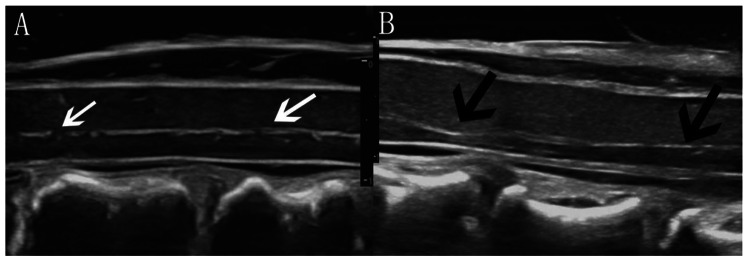
Location of the SCCEC in the SC. (**A**) SCCEC (linear hyperecho) was located almost exclusively in the center of the SC (white arrow). (**B**) Distinct forward movement of the SCCEC, especially in the CA (black arrow). The distance from 0 to 1 on the scale represents 1 centimeter

The SCCEC could be clearly displayed on IOUS and used to divide the SC into ventral and dorsal parts [14], with nearly the same thickness on both sides. We found that the SCCEC might shift toward the ventral side to varying degrees in different patients, especially in CAs. The forward movement rate (FMR) of the SCCEC was calculated as follows: FMR = dorsal thickness/the entire thickness of the SC in the same segment (Fig. 5). The larger the value, the more obvious the forward movement. The SCCEC FMRs in the CA and NCA were compared, and the correlation between the FMR in the CA and preJOA score was explored.

### Statistical analysis

SPSS 24.0 (IBM Corp.) was used for statistical analysis. All values are presented as the means ± standard deviations. The power of the study was based on the standard deviation (SD) of the JOA scores. The SD of the JOA scores was 2.68. Based on these differences, a sample size of 13 patients per group was required, with a power (1-β) of 80% and a type I error (α) of 5%. One-way ANOVA was used to explore the correlations between Area_free_ and sex; differences in preJOA scores between the homogeneous and heterogeneous groups; between the pulsation and nonpulsation groups; among the three different decompression statuses; and among the different compression positions, as well as differences in the SCCEC FMR and SCPA between the CA and NCA. Pearson correlation analysis was used to analyze the relationships between Area_free_ and age, as well as between the preJOA score and SCCR, HEA, and SCPA in the CA. Spearman correlation analysis was used to analyze the correlations between Area_free_ and height, weight, and BMI, as well as between the preJOA score and the SCCEC FMR in the CA. *P* < 0.05 was considered to indicate statistical significance.

## Results

From October 2019 to March 2022, 46 patients with multilevel DCM (≥ 3) confirmed by MRI who underwent FDL in the Department of Orthopedics of our hospital were included in this study. Four patients with a history of spinal surgery, 1 patient with a spinal deformity, 1 patient without enough clear sonograms, and 2 patients with partial loss of sonograms were excluded. Ultimately, 38 patients were successfully enrolled. There were 30 males and 8 females, and the mean age was 57.05 ± 10.29 (27–75) years. The basic information and clinical data of the patients are shown in Table 1.

### The relationship between spinal cord thickness and individual characteristics

Area_free_ was 74.62 ± 19.93 mm² (46–99 mm²), there was no significant correlation between Area_free_ and sex (*r* = 0.052; *p* = 0.822), height (*r*=-0.033; *p* = 0.844), weight (*r* =-0.081; *p* = 0.643) or BMI (*r* =-0.029; *p* = 0.867); however, Area_free_ was negatively correlated with age (*r* = 0.286; *p* = 0.041, *p* < 0.05). (Table 2)

**Table 2:** Correlations between basic data and Area_free_

Indicator	*r*	*P* value
Sex	0.052	*p* = 0.822
Height	-0.033	*p* = 0.844
Weight	-0.081	*p* = 0.643
BMI	-0.029	*p* = 0.867
Age	**-0.441**	***p*** = 0.006*

r = Correlation coefficient * Bold font denotes statistical significance, *p* < 0.05

### Correlation between the degree of deformation (compression rate) in CAs and the preJOA score

Area_compressed_ was 65.65 ± 16.07 mm² (26–93 mm². The SCCR, defined as Area_compressed_/Area_free_, was 0.87 ± 0.09 (0.54–0.98). There was no significant association between the SCCR and preJOA score (*r* =-0.025; *p* = 0.881). (Table 3)

**Table 3:** Correlations between IOUS indices and the preJOA score

Indicator	Results	*r*, *p* values
SCCR	0.87 ± 0.09	0.025; 0.881
HEA	0.314 ± 0.360 cm^2^	-0.334; 0.02* ***P*** **<0.05**
SCPA	0.39 ± 0.14 mm	-0.069; 0.339
SCCEC FMR	0.61 ± 0.09 mm	0.286; 0.041* ***P*** **<0.05**

r = correlation coefficient * Bold font denotes statistical significance, *p* < 0.05

### Differences in preJOA score between the homogeneous and heterogeneous groups and the relationship between preJOA score and HEA

Among the 38 patients, 23 patients were in the heterogeneous group, while 15 patients were in the homogeneous group. Individuals in the heterogeneous group had at least one abnormal echoic area in their SC. The preJOA score in the heterogeneous group was 9.71 ± 2.68, while that in the homogeneous group was 12.33 ± 2.48. The preJOA score was significantly lower in the heterogeneous group (*P*< 0.05; *p* = 0.005) (Table 4). In the heterogeneous group, the HEA was 0.314 ± 0.360 cm², and the HEA was negatively correlated with the preJOA score (*r* =-0.334; *p* = 0.02) (Table 3).

**Table 4:** Differences in the preJOA score according to different classifications

**Classification**	**Sample**	**PreJOA score**	***P*** **value**
All patients	38	10.75 ± 2.87	-
**Parenchymal echo** Heterogeneous	23	9.71 ± 2.68	P< 0.05 (p=0.005)* -
Homogeneous	15	12.33 ± 2.47	-
**Pulsation or not**			*P*＞0.05（p=0.303）
Pulsation	33	12.00 ± 3.65	-
No pulsation	5	10.56 ± 2.75	-
**Decompression status**			*P*＞0.05（P=0.414）
Type 1 (continued separation)	12	10.79 ± 3.12	-
Type 2 (contact and separation)	23	10.45 ± 2.86	-
Type 3 (continued contact)	3	12.83 ± 1.25	-
**Compression position**			*P*＞0.05（P=0.303）
Central	33	12.00 ± 2.73	-
Lateral	5	10.56 ± 2.88	-

* Bold font denotes statistical significance, *p* < 0.05

### Differences in preJOA score between the pulsation and nonpulsation groups, differences in the SCPA between the CA and NCA, and the association between the SCPA in the CA and the preJOA score

Thirty-three patients were categorized into the SC pulsation group; the remaining 5 patients were categorized into the nonpulsation group. The corresponding preJOA scores were 12.00 ± 3.65 and 10.56 ± 2.75, respectively, with no significant difference between the two groups (*P* > 0.05; *p* = 0.303) (Table 4). The SCPA in the most severe CA and adjacent NCA were 0.39 ± 0.14 mm and 0.18 ± 0.08 mm, respectively, exhibiting a significant difference (*P* < 0.001; *p* = 0.000) (Table 5). There was no association between SCPA in the CA and preJOA score (*r*=-0.069, *P* > 0.05; *p* = 0.339) (Table 3).

**Table 5:** Differences in the SCPA and SCCEC FMR between the CA and NCA

Indicator	CA	NCA	*P* value
SCPA	0.39 ± 0.14 mm	0.18 ± 0.08 mm	***P*** ** < 0.001 (** ***p*** = 0.000)**
SCCEC FMR	0.61 ± 0.09 mm	0.56 ± 0.05 mm	***P*** ** < 0.05 (** ***p*** = 0.007)*

*Bold font denotes statistical significance, *p* < 0.05

### Differences in the preJOA score among three different decompression statuses

There were 12 patients in the continuous separation group, 23 patients in the contact and separation group, and 3 patients in the continued contact group. The corresponding preJOA scores were 10.79 ± 3.12, 10.45 ± 2.86, and 12.83 ± 1.25, respectively, with no significant difference in the preJOA score among the three types of contact (*P* > 0.05; *p* = 0.414) (Table 4).

### Differences in the preJOA score among different compression positions

Thirty-three patients were allocated to the central compression group, and 5 patients were allocated to the lateral compression group. The corresponding preJOA scores were 12.00 ± 2.73 and 10.56 ± 2.88, respectively. The difference in the preJOA score between the two groups was not statistically significant (*P*>0.05; *p* = 0.303) (Table 4).

### Differences in the SCCEC FMR between the CA and NCA and the relationship between the preJOA score and SCCEC FMR in the CA

The SCCEC FMR was 0.61 ± 0.09 in the CA and 0.56 ± 0.05 in the NCA, with a significantly more pronounced forward movement in the CA (*P* < 0.05; *p* = 0.007) (Table 5). Furthermore, there was a mild positive correlation between the FMR in the CA and the preJOA score (*r* = 0.286, *p* < 0.05; *p* = 0.041) (Table 3).

## Discussion

IOUS has been applied in cervical laminoplasty for patients with DCM for a long time, and the existing studies have mainly focused on aspects such as spinal cord morphology, echogenicity, and pulsation. For surgeons and sonographers, quickly interpreting ultrasound images to obtain useful information is highly important; therefore, the main purpose of this study was to systematically and comprehensively explore the IOUS characteristics of patients with DCM. In addition to observing the morphology, internal echo and pulsation of the SC, the literature has focused on the SC decompression status, compression position, and a special structure of the SC, SCCEC, which can be observed only on IOUS and cannot be observed on MRI [10, 14, 19, 23–25]. Therefore, this study discusses the above six parts. In addition to focusing on SC deformation in the CAs, large individual differences in normal SC thickness have also been observed in the NCAs.

The results showed that among patients aged 27 to 75 years old, the SC was thinner in older patients, and the SCCR was not related to the severity of the disease. In terms of echogenicity, uneven echogenicity indicated more serious disease, and this disease was positively correlated with HEA. In terms of pulsation, the presence or absence of pulsation did not seem to be an indicator of the severity of the disease, and the pulsation of the SC in CAs was most obvious. After decompression, neither the SC decompression status nor the compression position was related to the severity of the disease. For the location of the SCCEC, the more obvious the FMR was, the milder the symptoms were, and the FMR was more obvious in CAs than in NCAs.

First, in terms of SC morphology, the study included mainly the relationship between normal SC thickness and individual differences and the relationship between SCCR and disease severity. Recent research on SC thickness has focused mainly on animal experiments [34] and human postmortem studies [35]. Due to the atrophy bias of the SC in cadavers, this metric cannot completely reflect the real thickness of the SC in living organisms, and limited research has been conducted on the thickness of the SC in vivo via MRI and CT imaging [36, 37]. Compared with traditional imaging modalities, the thickness of the SC is not affected by the morphology of the spinal canal on IOUS; thus, the SC thickness could be used to realize real-time dynamic imaging and perfectly present the true state of the SC. To the best of our knowledge, this is the first study in which correlations between SC thickness and individual factors were explored using IOUS. Our results are consistent with the results of previous studies, and the results showed large individual differences in the cross-sectional area of the SC. In adults (≥ 18 years old), the thickness of the SC was negatively correlated with age but not with sex [35]. In that paper, SC thickness was significantly positively correlated with body height, which was different from our results. In our study, SC thickness was not correlated with height, weight, or BMI. This difference might be related to the use of different research methods and small sample sizes. However, further research is necessary to determine whether body height is related to SC thickness.

Although the SC in the CA was completely decompressed during the operation, the SC still showed varying degrees of deformation. studies have reported no significant correlation between compression indices and disease severity on IOUS [22, 33], but the relationship on preoperative MRI remains disputed [38, 39]. Considering that the compression of SC is uneven, in this study, the ratio of the compression area was used instead of the ratio of the compression diameter, which could more accurately reflect the deformation of the SC after decompression. Surprisingly, as shown in previous studies, the ratio of the compression area was also not associated with preoperative disease severity [31]. Despite severe SC deformation after decompression, this observation could not indicate the relative severity of the patient’s condition and might instead be related to the complex pathophysiological mechanisms of DCM. One possible explanation is that mechanical compression can trigger SC injury, but the real cause of injury is secondary pathological injury caused by static compression, such as edema, ischemia, SC atrophy, neuronal apoptosis, and cystic necrosis [40–43].

Second, there were echo changes in the spinal parenchyma. In the present study, 23 of the 38 patients (61%) exhibited abnormal echoes in the spinal parenchyma, which mainly manifested as more than one hyperechoic region of different sizes and shapes in the SC; among them, anechoic regions could be observed in the hyperechoic region in 2 patients. Interestingly, not all of the hyperechoic regions were in the compression zone, and some were in a nearby area (Fig. 2C). This finding is also consistent with the above explanation suggesting compression as the initiating factor; the areas where the pathological changes were obvious were not necessarily the areas where the compression was the most severe. Chronic compression can lead to the loss of capillary endothelial cells, neurocytes, and proliferative fibroblasts as well as fibrin deposition and even fibrosis in the compressed region. These possible pathological changes may eventually contribute to an uneven density in the SC [44], which could in turn lead to uneven echoes on ultrasound images according to ultrasonography principles [45], with significantly different densities in adjacent tissues resulting in increased echoes [22]. Therefore, echo changes in the SC parenchyma can be observed during IOUS.

Previous studies have reported that hyperechogenicity might predict postoperative neurological function [22, 33], however, in this study, we evaluated the correlation between changes in SC parenchymal echoes and the severity of preoperative symptoms. Unlike in previous studies, we did not quantify the gray value [10, 32] but rather the HEA. High-resolution ultrasound can quickly and accurately reveal whether there is an abnormal echo in the SC. The results showed that patients with abnormal echoes had more severe symptoms, and the HEA was positively correlated with the preoperative severity of the disease. Thus, the HEA could assist surgeons in quickly recognizing and understanding the state of the SC.

Third, regarding the state after decompression, many previous studies have classified the decompression state into three types—continuous separation, contact separation, and continuous contact—according to the relationship between the anterior border of the SC and the compression elements [24, 25, 31]. In the continuous contact type, the disappearance of cerebrospinal fluid in the subarachnoid space in front of the SC, which might affect the circulation of cerebrospinal fluid and the blood supply of the SC and nerve roots, is considered a significant indicator of inadequate decompression [23, 46]. Nevertheless, surgeons have no direct view of the anterior or internal structures of the SC during conventional posterior decompression. These findings emphasize the value of IOUS in posterior decompression for DCM by virtually eliminating possible residual compression.In this study, 3 patients were classified as having continuous contact and underwent further decompression during the operation. Under IOUS guidance, surgeons could control the degree of redecompression. Excessive decompression can lead to secondary instability and segmental kyphosis [9], while inadequate decompression can lead to symptom recurrence. The results of this study showed that there was no obvious correlation between disease severity and decompression status. Therefore, when observing decompression status, greater attention should be given to the presence of cerebrospinal fluid in the CA.

Fourth, SC pulsation can only be detected by IOUS. IOUS showed that the SCs of different patients exhibit varying degrees of pulsation or no pulsation, and the SCPA in the same patients differs between the CA and NCA. Previous research has shown that the movement pattern and amplitude of the SC are not correlated with postoperative neurological function recovery [47, 48]. In this study, 33 patients were categorized into the SC pulsation group, and 5 patients were categorized into the nonpulsation group. Our findings revealed no difference in preoperative symptoms between the pulsation group and the nonpulsation group, and the pulsation amplitude in the CA was not related to the severity of the disease, nevertheless, the pulsation amplitude in the CA was significantly greater than that in the adjacent NCA. In fact, as early as 1984, Jokich et al. described SC pulsation as a complex phenomenon related to heart rate, breathing, dural pulsation and other factors, among which the transmitted pulsations from compressed spinal arteries play a major role [8]. Since the force dissipates into the cerebrospinal fluid, the pulsation amplitude near the CA is weakened. Interestingly, similar to the findings reported by Jokich, we found that the SC pulsations were most pronounced in the areas with the most intense compression, while the pulsations decreased significantly in the adjacent NCAs. The relationship between SC pulsation and SC function remains controversial [8, 49, 50], and additional research is needed.

Fifth, regarding the position of the anterior compression of the SC, previous studies have shown that chronic compression reduces regional SC blood flow, and local deformation leads to further ischemia and eventually to impaired SC perfusion, which has long been considered a central pathophysiological tenet of DCM [44, 51]. The SC is supplied almost exclusively by the anterior spinal cord artery (ASCA), which traverses the anterior median sulcus of the cord [52, 53] and is easily subjected to extrinsic compression by spondylotic ridges and other hypertrophic connective tissue. Patients were divided into central and lateral compression groups according to whether the ASCA was compressed. In the axial view, patients whose SC was compressed by anterior elements in the central region were classified as having central compression, in which the ASCA was likely to be compressed, while the probability of the ASCA being compressed was lower in those classified as having lateral compression. This classification is somewhat different from that of previous classifications [23], in which central compression was further divided into whole width and compressed only in the central part of the SC; however, the results are somewhat similar. In theory, the ASCA is more vulnerable to central compression, and preoperative symptoms are more severe; however, our results showed no significant difference in preoperative symptoms between the two groups. This finding may be related to the complexity of the pathogenesis of DCM, and due to the small sample size, we did not stratify patients by the course of disease or the degree of compression. The correlation between ASCA compression and SC function can be further verified in animal experiments.

Sixth, SCCEC, a special intramedullary structure displayed easily on IOUS but cannot be detected on conventional MRI or CT, currently presents as a linear hyperechogenic area in the middle to ventral part of the SC on IOUS [12, 14]. Few reports on SCCEC exist in the literature. A combination of sonography and histoanatomical examinations revealed that SCEC is produced by the interface between the myelinated ventral white commissure and the central end of the anterior median fissure and is usually centered on the midline of the SC but might lie slightly anterior to the center point of the SC in the cervical SC [54]. In 2022, our team reported the predictive value of the transverse diameter of the SCCEC for postoperative neurological function recovery [14]. To our knowledge, there have been no reports on SCCEC movement forward. In this study, we observed that the SCCEC was not centrally located in any of the patients on the sagittal plane but moved forward to the ventral side to varying degrees. Therefore, we proposed the concept of the forward movement rate (FMR) of the SCCEC, and we found that the FMR in the CA was significantly greater than that in the NCA. However, there was a slight positive correlation between the FMR and the preJOA score; in other words, the more the SCCEC moved forward, the less severe the preoperative symptoms were. This was a very interesting discovery; this seemingly contradictory conclusion, in addition to the error caused by the small sample size of this study, also provides a new idea and direction for future research on SCCEC. In a follow-up study, we will focus on the possible causes of this phenomenon and further explore the correlation between the FMR and postoperative neurological recovery.

Our study has the following limitations. First, as an observational study, the sample size was relatively small. Second, the preJOA score was the only preoperative neurological function indicator that might be affected by doctor‒patient subjectivity and could be biased. Third, due to the limitations of the surgical incision, the scan window was relatively small, and the SC segments could not be located as accurately as on MRI. Additionally, for several parameters, such as areas of hyperechogenicity in the SC, we quantified only the area, not the intensity.

## Conclusions

In adults, the cross-sectional area of the SC decreases with age. The SCCEC FMR and SCPA are more pronounced in CAs. The degree of SC deformation, pulsation, decompression status and compression type were not significantly related to the severity of the disease. Surgeons should pay more attention to changes in spinal parenchymal echoes, the position of the SCCEC and whether decompression in front of the SC is sufficient. In the future, it is necessary to expand the sample size and carry out multi-center studies as far as possible to conduct systematic and comprehensive analysis of ultrasonography characteristics of patients with DCM, so that surgeons can obtain more useful information quickly by IOUS, and at the same time sonographers can break through the visual blindness of the spinal canal and observe the internal structure of the spinal canal, so as to provide an objective theoretical basis for further exploration of the pathogenesis of DCM.

### Electronic supplementary material

Below is the link to the electronic supplementary material.

Video 1. The SC pulsation group

Video 2. The SC nonpulsation group

Video 3. Continued separation: The SC completely floated in the cerebrospinal fluid

Video 4. Contact and separation: As the SC pulsated, the anterior edge contacted and separated from the anterior compression element

Video 5. Continued contact: The anterior compression element and the anterior edge of the SC were in constant contact

## Data Availability

The datasets used or analyzed during the current study are available from the corresponding author on reasonable request.

## Declarations

### Key results

In this retrospective study of 38 patients, the cross-sectional area of the SC was negatively correlated with age. The preJOA score was significantly lower in the heterogeneous group than in the homogeneous group. The HEA was negatively while the SCCEC FMR was positively correlated with the preJOA score. The SCCEC FMR and SCPA in CAs were significantly greater than those in NCAs.

## Abbreviations

IOUS: Intraoperative ultrasound
DCM: Degenerative cervical myelopathy
FDL: French-door laminoplasty
SC: Spinal cord
Area_free_: Cross-sectional area of the noncompressed area
Area_compressed_: Cross-sectional area of the compressed area
SCCR: Spinal cord compression rate
HEA: Hyperechoic area
SCPA: Spinal cord pulsation amplitude
SCCEC: Spinal cord central echo complex
FMR: Forward movement rate
CA: Compressed area
NCA: Noncompressed area
preJOA: Preoperative Japanese Orthopedic Association score
ASCA: Anterior spinal cord artery
